# Fine‐tuning PERK signaling for neuroprotection

**DOI:** 10.1111/jnc.14112

**Published:** 2017-08-08

**Authors:** Mark Halliday, Daniel Hughes, Giovanna R. Mallucci

**Affiliations:** ^1^ MRC Toxicology Unit Leicester UK; ^2^ Department of Clinical Neurosciences University of Cambridge Cambridge Biomedical Campus Cambridge UK; ^3^ UK Dementia Research Institute at University of Cambridge Island Research Building Cambridge Biomedical Campus Cambridge UK

**Keywords:** Alzheimer's disease, neurodegeneration, neuroprotection, therapeutics, unfolded protein response

## Abstract

Protein translation and folding are tightly controlled processes in all cells, by proteostasis, an important component of which is the unfolded protein response (UPR). During periods of endoplasmic reticulum stress because of protein misfolding, the UPR activates a coordinated response in which the PERK branch activation restricts translation, while a variety of genes involved with protein folding, degradation, chaperone expression and stress responses are induced through signaling of the other branches. Chronic overactivation of the UPR, particularly the PERK branch, is observed in the brains of patients in a number of protein misfolding neurodegenerative diseases, including Alzheimer's, and Parkinson's diseases and the tauopathies. Recently, numerous genetic and pharmacological studies in mice have demonstrated the effectiveness of inhibiting the UPR for eliciting therapeutic benefit and boosting memory. In particular, fine‐tuning the level of PERK inhibition to provide neuroprotection without adverse side effects has emerged as a safe, effective approach. This includes the recent discovery of licensed drugs that can now be repurposed in clinical trials for new human treatments for dementia. This review provides an overview of the links between UPR overactivation and neurodegeneration in protein misfolding disorders. It discusses recent therapeutic approaches targeting this pathway, with a focus on treatments that fine‐tune PERK signaling.

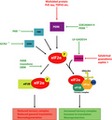

**Abbreviations used**
Aβ: amyloid beta
AD: Alzheimer's disease
ALS: amyotrophic lateral sclerosis
ARE: antioxidant response element
ATF4: activating transcription factor 4
ATF6: activating transcription factor 6
CREB: cAMP response element binding protein
ER: endoplasmic reticulum
ERAD: ER‐associated decay
ERSE: ER stress response element
FTD: frontotemporal dementia
GSK3β: glycogen synthase kinase 3β
HD: Huntington's disease
IRE1: inositol requiring enzyme 1
MSA: multiple systems atrophy
NFT: neurofibrillary tangles
Nrf2: nuclear factor (erythroid‐derived)‐like 2
PD: Parkinson's disease
PERK: protein kinase RNA (PKR) like ER kinase
PSP: progressive supranuclear palsy
RIDD: regulated IRE1‐dependent decay
UPR: unfolded protein response
XBP1s: X‐box binding protein 1 spliced

The protein misfolding neurodegenerative diseases are increasingly described by their biochemical similarities, including the aggregation of misfolded proteins, synaptic dysfunction, mitochondrial and endoplasmic reticulum (ER) stress, rather than by neuropathological differences in affected brain regions, symptoms and disease progression. Thus, neurodegenerative diseases including Alzheimer's (AD), Parkinson's (PD), Huntington's (HD), amyotrophic lateral sclerosis (ALS), prion disease, frontotemporal dementia (FTD) and progressive supranuclear palsy (PSP) are all characterized by the presence of disease‐specific misfolded proteins, where the identity and localization of the misfolded protein is associated with the unique pathophysiology of each disease. As a result of the failure of a number of human therapeutics targeting specific misfolded proteins, notably the disappointments of several recent trials of amyloid beta (Aβ)‐reducing therapies in Alzheimer's disease (Doody *et al*. 2013, 2014; Abushouk *et al*. 2017), researchers are increasingly pursuing other avenues for future therapeutic targets (Morris *et al*. 2014). Thus, studying the processes and downstream effects of protein misfolding generally has become a fruitful area of research in this respect.

Proteins control our most vital cellular functions, and a protein's proper functioning is dependent upon its correct three‐dimensional structure. Unsurprisingly, the process of forming these complex tertiary and sometimes quaternary conformations is susceptible to errors (Dill and MacCallum 2012). This is compounded by the correct folding of a protein ‘competing’ with its innumerable potential misfolded states. Furthermore, many misfolded proteins involved in familiar cases of neurodegenerative disease contain mutations that destabilize the correct folding or stabilize a misfolded state. In the crowded environment of the cell, proteins must maintain their correct conformation while being constantly buffeted by collisions with neighboring proteins (Ellis and Minton 2006). Protein folding also occurs in several distinct compartments in the cell, from specialized organelles such as mitochondria and peroxisomes, to the large compartments of the ER for membrane and secreted proteins. The different chemical composition of each of these compartments results in different protein‐folding issues that each cell must address and prevent. It is unsurprising that these strict requirements and external influences mean many proteins do not achieve their correct conformations, leading to cell death and disease. This is especially serious in post‐mitotic neurons that cannot be replaced.

Like all cells, neurons have regulatory systems in place for protein quality control. Chaperones are expressed constitutively and further induced in response to the accumulation of unfolded proteins. The ER is a major site of cellular protein synthesis, synthesizing around a third of the proteome. A high proportion of these proteins are secretory, transmembrane and organelle targeted proteins. (Braakman and Bulleid 2011). The main protein quality control mechanism in the ER is the unfolded protein response (UPR), which is activated if the protein folding homeostasis of the ER is disturbed. Its role is to detect and remove misfolded or problematic proteins, and to prevent aggregation as early as possible. When the misfolded proteins cannot be properly refolded, pathways such as autophagy, regulated IRE1‐dependent decay (RIDD) and ER‐associated degradation (ERAD), are engaged (Nedelsky *et al*. 2008; Smith *et al*. 2011).

However, in the protein misfolding neurodegenerative diseases, there is a failure in one or multiple of these protein quality control systems (Sweeney *et al*. 2017). This review will focus on the normal function of the UPR, its dysfunction in neurodegenerative disease, and recent advances in determining the level of fine‐tuning needed to modulate the UPR as a treatment for neurodegeneration safely and effectively.

## The unfolded protein response (UPR)

**Figure 1:**
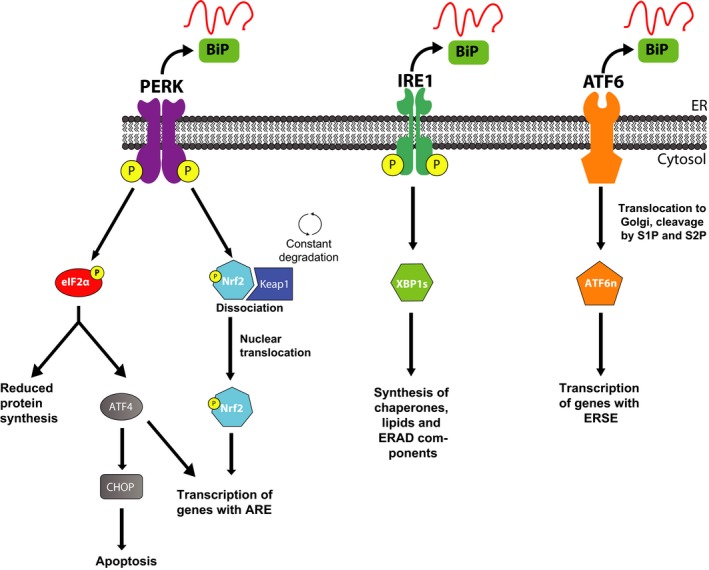
The unfolded protein response. The presence of misfolded proteins causes the dissociation of BiP from the three unfolded protein response (UPR) sensors, protein kinase RNA (PKR) like ER kinase (PERK), IRE1 and activating transcription factor 6 (ATF6), leading to their activation. PERK phosphorylates eIF2α, leading to translational repression and the up‐regulation of a subset of UPR target genes including activating transcription factor 4 (ATF4) and the pro‐apoptotic CHOP. PERK also phosphorylates nuclear factor (erythroid‐derived)‐like 2 (Nrf2), leading to its dissociation from the inhibitory Keap1. Nrf2 is then free to activate genes with an ARE involved with the redox/antioxidant response. IRE1 activation leads to the splicing of X‐box binding protein 1 spliced (XBP1), which then activates many UPR target genes related to protein folding, lipid synthesis, protein translocation into the ER and ER‐associated decay (ERAD). ATF6 activation targets genes under the control of an ER stress response element (ERSE), including molecular chaperones (such as BiP), folding enzymes and components of the ERAD system.

The UPR is the cells main defense against the build‐up of misfolded proteins, which acts to restore proteostasis during periods of ER stress. It achieves this by enacting a series of transcriptional and translational changes that increase the folding and degradative capacity of the ER (Walter and Ron, 2011). The UPR is controlled by three transmembrane endoplasmic reticulum proteins, protein kinase RNA (PKR)‐like ER kinase (PERK), inositol‐requiring enzyme 1 (IRE1) and activating transcription factor 6 (ATF6) (Fig. 1).

**Figure 2:**
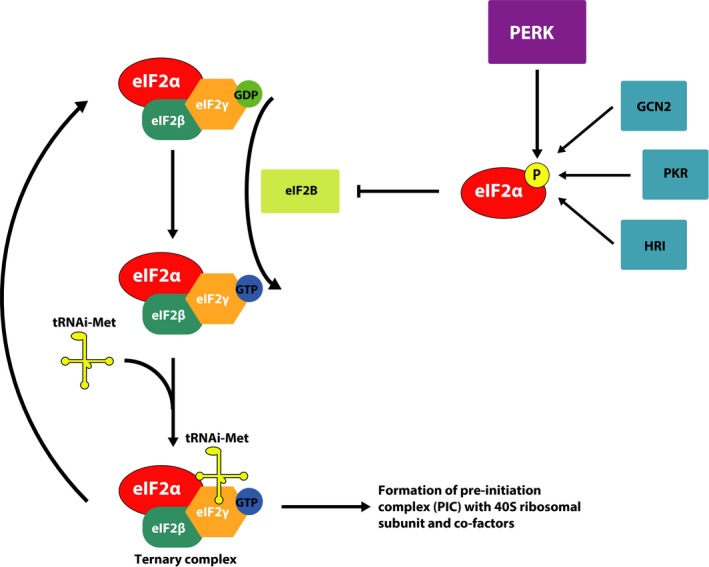
The unfolded protein response (UPR) and ISR converge on eIF2α phosphorylation, leading to a reduction in ternary complex. Protein kinase RNA (PKR) like ER kinase (PERK), PKR, GCN2 and HRI all phosphorylate eIF2α in response to a variety of stresses. Under normal conditions, the three subunits of eIF2 form ternary complex with methionine transfer RNA and GTP supplied by the guanine exchange factor eIF2B. This complex is then loaded into the ribosome with the mRNA to be translated, forming the pre‐initiation complex. When eIF2α is phosphorylated, it binds tightly to eIF2B, preventing it from exchanging GDP to GTP. This prevents the formation of ternary complex and potently inhibits the initiation of translation.

Of the three arms of the unfolded protein response, the PERK/eIF2α axis is the most strongly linked to neurodegenerative disease. The PERK monomer sits within the ER membrane: its luminal N‐terminus binds to BiP (GRP78) when inactive, while its cytoplasmic C‐terminal contains its kinase domain (Cui *et al*. 2011). BiP is a chaperone protein that holds PERK, along with IRE‐1 and ATF6, in their inactive states. It has a relatively low but wide ranging affinity for the hydrophobic regions of misfolded proteins, allowing it to recognize and bind a spectrum of misfolded species. PERK is activated on BiP dissociation, this dissociation allows dimerization and autophosphorylation, to form activated PERK‐P. Activated PERK in turn phosphorylates the α subunit of eukaryotic initiation factor eIF2 at ser51, which rapidly and potently inhibits the initiation of translation, effectively shutting down the majority of protein synthesis (Harding *et al*. 1999). Translational initiation involves the formation of ternary complex (consisting of eIF2, initiator methionine transfer RNA and guanosine triphosphate (GTP), supplied by eIF2B), that loads the mRNA to be translated into the ribosome. When eIF2α is phosphorylated, it binds tightly to eIF2B, preventing it from loading GTP into the ternary complex (Fig. 2). The crystal structure of eIF2B and its binding to eIF2 has recently been solved (Kashiwagi *et al*. 2016). Because of the relative scarcity of eIF2B compared to eIF2α, even low amounts of eIF2α‐P can have potent inhibitory effects on translation rates (Spriggs *et al*. 2010). However, not all translation is ceased after PERK/eIF2α‐P activation, select mRNA transcripts are induced because of the presence of upstream open reading frames in their 5′UTRs that causes them to be preferentially translated during periods of low ternary complex (Harding *et al*. 2000; Lu *et al*. 2004). One such protein is activating transcription factor 4 (ATF4), which induces the expression of a host of proteins involved with cellular metabolism, redox homeostasis and apoptosis (Blais *et al*. 2004; Vattem and Wek 2004). Under physiological conditions, this interruption in protein translation allows a stressed cell to resolve the cause of the stress and resume proper protein folding. Once this occurs eIF2α‐P is dephosphorylated by GADD34, the regulatory subunit of the phosphatase PP1 (Novoa *et al*. 2001). However, if the cellular stress cannot be resolved, ATF4 induces the expression of the pro‐apoptotic C/EBP homologous protein (CHOP) (Fawcett *et al*. 1999). The timing or regulatory signals that determine the switch between the PERK induced protection from mild ER stress and to the CHOP controlled switch to pro‐death signaling are not understood, but are extremely important in the context of neurodegeneration.

PERK‐P also phosphorylates nuclear factor (erythroid‐derived 2)‐like 2 (Nrf2), a bZip transcription factor involved in the regulation of a variety of antioxidant genes that are regulated by an antioxidant response element (Itoh *et al*. 1997; Alam *et al*. 1999). In unstressed cells, Nrf2 is maintained in an inactive state by Keap1 (Itoh *et al*. 1999), but ER stress induces a PERK dependent dissociation of the Nrf2/Keap1 complex, allowing nuclear translocation (Cullinan *et al*. 2003). This pro‐survival response is independent of eIF2α‐P (Cullinan *et al*. 2003), and thus places PERK at the center of two distinct patterns of gene expression mediated by the direct phosphorylation of eIF2α and Nrf2.

The most conserved UPR signaling branch is initiated by IRE1. Dimerization of IRE1 and its autophosphorylation activates its endoribonuclease activity to catalyze the splicing of transcription factor X‐box binding protein 1 (XBP1) mRNA (Calfon *et al*. 2002). This event removes a 26‐nucleotide intron, causing a frame shift in the reading frame of the mRNA. Spliced XBP1 (XBP1s) is a long‐lived and potent transcription factor that controls a large number of UPR target genes related to protein folding, lipid synthesis, protein translocation into the ER, ERAD and other processes (Hetz *et al*. 2011). IRE1 activation also induces degradation of the mRNAs of ER incoming proteins through RIDD to reduce the folding load inside the ER (Maurel *et al*. 2014). IRE1 also activates stress kinases, such as the apoptosis signal‐regulating kinase 1 pathway and JUN amino‐terminal kinase (Coelho and Domingos 2014). Interestingly, IRE1 can be directly activated by unfolded proteins (Gardner and Walter 2011).

The potent transcription factor ATF6 is also anchored to the ER membrane by BiP in unstressed cells. Upon ER stress, dissociation of BiP unmasks a Golgi localization signal (Shen *et al*. 2002). Upon arrival at the Golgi, ATF6 is cleaved by site 1 and 2 proteases (Ye *et al*. 2000), freeing a cytosolic ATF6 fragment termed ATF6n. This translocates to the nucleus to activate the transcription of genes encoding ER resident proteins that participate in ER quality control processes. ATF6n targets genes under the control of an ERSE including molecular chaperones (such as BiP), folding enzymes and components of the ERAD system (such as degradation in ER protein 3, derlin‐3) for disposal of misfolded proteins (Adachi *et al*. 2008).

There is much crosstalk between the different arms of the UPR (Brewer 2014). ATF6 can induce the activation of CHOP, and ATF4 can induce IRE1 activation and XBP1 splicing (Yoshida *et al*. 2000; Tsuru *et al*. 2016). ATF4 and Nrf2 can bind DNA as a homodimer or as a heterodimer, as well as other bZip transcription factors to regulate different gene sets. CHOP works in concert with ATF4 to activate expression of GADD34, creating a negative feedback loop that limits translational repression (Ma and Hendershot 2003); however, induction of GADD34 seems to fail during neurodegenerative disease, leading to chronic UPR activation (Moreno *et al*. 2012).

## The integrated stress response (ISR)

Other signaling pathways also converge on eIF2α, leading to its phosphorylation and subsequent downstream translational repression. PKR phosphorylates eIF2α in response to double stranded RNA introduced by viral infection, preventing translation of viral mRNAs, and also promotes apoptosis in terminally infected cells (Galluzzi *et al*. 2008). PKR can also be activated during periods of oxidative or ER stress (Onuki *et al*. 2004; Nakamura *et al*. 2010), as well as cytokine signaling and growth factors (Cheshire *et al*. 1999), demonstrating a general role for PKR in cellular physiology. PKR induces expression of the pro‐apoptotic transcription factor CHOP in response to conditions such as hypoxia (Lozon *et al*. 2011), and ER stress (Li *et al*. 2010). GCN2 is primarily a sensor of amino acid availability and a regulator of changes in gene expression in response to amino acid deprivation (Deval *et al*. 2009). To date, only eIF2α has been characterized as a GCN2 target. It is also activated by glucose deprivation (Ye *et al*. 2010), viral infection (Berlanga *et al*. 2006) and UV irradiation (Grallert and Boye 2007). A significant proportion of the genes targeted for up‐regulation by ATF4 are involved in amino acid import and metabolism (Harding *et al*. 2003). GCN2 is highly expressed in the brain, where it is believed to be involved in behavioral adaptation to diets deficient in amino acids (Maurin *et al*. 2005). The final known eIF2α kinase is heme‐regulated eIF2α kinase (HRI). It couples the expression of globin genes to the amount of heme present in the cell, promoting the survival of erythroid precursors when iron levels are low (Chen 2007). It has also been implicated in responses to proteasome inhibition (Yerlikaya *et al*. 2008), although the mechanisms via which this occurs are unclear. Little is known about any neuronal effects of HRI activation.

## UPR dysfunction in human neurodegenerative disease

A growing body of evidence is implicating the UPR in the pathology of the protein misfolding neurodegenerative diseases. Recent *in vivo* data from multiple mouse models in the context of histopathological studies in human disease provide potential evidence linking UPR activation to neurodegenerative disease.

Alzheimer's disease is characterized by two classic neuropathological hallmarks: neurofibrillary tangles comprised of intracellular aggregates of phosphorylated tau, and extracellular plaques that contain aggregates of Aβ. Markers specific for UPR activation, such as PERK‐P, eIF2α‐P, IRE1‐P and BiP, are increased in AD brain tissue (Chang *et al*. 2002; Hoozemans *et al*. 2005, 2009; Unterberger *et al*. 2006; Stutzbach *et al*. 2013). The levels of BiP, PERK‐P and IRE1‐P in AD neurons correlate with the observed Braak stages, a neuropathological method for determining the levels neurofibrillary tangles and phosphorylated tau (Hoozemans *et al*. 2009; Duran‐Aniotz *et al*. 2017), indicating that the UPR is involved in AD pathology at an early stage.

The closely related tauopathies, such as PSP, FTD and Pick's disease, contain inclusions of filamentous tau with a greatly reduced or absent amyloid component. PERK‐P, eIF2α‐P and IRE1‐P have all been observed in affected brain areas in these tauopathies (Unterberger *et al*. 2006; Nijholt *et al*. 2012; Stutzbach *et al*. 2013). These UPR markers appear in the same neurons and glia that present with abnormal tau phosphorylation, again suggesting phosphorylated tau aggregates and activation of the UPR during neurodegeneration are closely linked. A genome‐wide association study searching for common variants influencing the risk of PSP identified a polymorphism in the PERK gene, EIF2AK3 (Hoglinger *et al*. 2011). Patient‐derived lymphoblastoid cell lines containing this polymorphism displayed an increased UPR stress response (Liu *et al*. 2012), suggesting that the variant leads to overactivation of the UPR, that leads to an increased risk of this tauopathy.

In prion diseases, rapidly progressive neuronal loss accompanies extracellular aggregation and accumulation of the misfolded form of the prion protein, termed PrP scrapie (PrP^Sc^), formed by recruitment and conversion of normal cellular prion protein (PrP^C^). Analysis of ER stress markers, particularly phosphorylated proteins, such as PERK‐P and eIF2α‐P, is difficult in human prion disease because of the numerous precautions needed when handling infected tissue, leading to long post‐mortem delays (Scheper and Hoozemans 2015) and have been reported as not being detected. However, increased levels of GRP58, GRP94 and BiP, along with caspase‐12 activation in cortical samples from both sporadic and variant Creutzfeldt‐Jacob disease cases have been observed (Hetz *et al*. 2003), along with protein disulfide isomerase which catalyses protein folding (Yoo *et al*. 2002).

In Parkinson's disease, there is a loss of dopaminergic neurons in a selective manner in the substantia nigra pars compacta and accumulation of α‐synuclein in eosinophilic cytoplasmic inclusions called Lewy bodies. PERK‐P and eIF2α‐P are observed in dysfunctional substantia nigra neurons (Hoozemans *et al*. 2007). Multiple system atrophy (MSA) is a rare neurodegenerative disease that shares similarities with PD, including the intracellular accumulation of α‐synuclein. PERK‐P, eIF2α‐P and IRE1‐P are increased in MSA cases (Makioka *et al*. 2010). These UPR components are observed to be closely associated with α‐synuclein containing cytoplasmic inclusions in glia cells, during the initial stages of the disease (Makioka *et al*. 2010). The relationship between ER stress markers and the aggregation of α‐synuclein in PD and MSA suggests a robust link between α‐synuclein and overactivation of the UPR. In *in vitro* models that over‐express wild‐type or mutant α‐synuclein, vulnerability to ER stress is increased, supporting this assertion (Stefanis *et al*. 2001; Cooper *et al*. 2006).

ALS is characterized by the death of motor neurons in the spinal cord, cortex and brain stem, and the aggregation of unfolded proteins that include SOD1, TDP‐43 and FUS. UPR signaling by PERK, ATF6 and IRE1 are increased in the spinal cord of sporadic ALS patients alongside caspase 4 (Atkin *et al*. 2008), as well as downstream signaling components such as ATF4, GRP58 and XBP1s (Hetz *et al*. 2009). Other immunohistochemical analyses have observed an increase in CHOP and BiP in the spinal cord of ALS patients (Ito *et al*. 2009).

In Huntington's disease, there is accumulation of intracellular protein aggregates of huntingtin containing long regions of polyQ repeats and selective neuronal death, predominantly in the striatum. Levels of BiP and CHOP mRNA are increased in the parietal cortex of HD (Carnemolla *et al*. 2009) and IRE1‐P and BiP is observed in striatal tissue of HD patients (Lee *et al*. 2012). A systems biology approach identified major alterations in UPR signaling in HD (Kalathur *et al*. 2015).

Animal models of many of these neurodegenerative disorders also display the hallmarks of ER or UPR stress. Raised levels of eIF2α‐P and PERK‐P are observed in prion‐diseased mice (Moreno *et al*. 2012, 2013), in mice expressing the FTD‐associated human tau mutation P301L (Abisambra *et al*. 2013; Radford *et al*. 2015), in mouse models of AD (Devi and Ohno 2014), in mutant SOD1‐expressing mice (Saxena *et al*. 2009; Wang *et al*. 2014) and in a mouse model of PD (Colla *et al*. 2012),

Despite being primary disorders of myelin, where axonal (and hence neuronal) loss is secondary to loss of myelin sheaths, several demyelinating diseases are also associated with UPR activation [see (Clayton and Popko 2016) for review]. Brain tissue from patients with vanishing white matter and Pelizaeus–Merzbacher diseases display UPR activation (Southwood *et al*. 2002; van der Voorn *et al*. 2005). Similarly, mouse models of Pelizaeus–Merzbacher, vanishing white matter diseases and Charcot–Marie–Tooth (CMT1B) peripheral neuropathy mouse models all display expression of UPR markers such as CHOP, ATF4 and XBP1s (van Kollenburg *et al*. 2006; Pennuto *et al*. 2008; Southwood *et al*. 2013). In CMT1B, UPR modulation is therapeutic (D'Antonio *et al*. 2013; Das *et al*. 2015). Mouse models of the dysmyelinopathy, X‐linked adrenoleukodystrophy, similarly show UPR overactivation (Launay *et al*. 2017).

## Linking UPR activation to neurodegeneration

The wealth of evidence showing the correlation between UPR activation and neurodegeneration in neuropathological samples is not in itself proof of causation. However, studies in animal models have provided much evidence in support of this. The first demonstration that chronic PERK/eIF2α‐P signaling drives neurodegeneration was seen in prion‐diseased mice (Moreno *et al*. 2012) (Table 1). They found that levels of PERK‐P and eIF2α‐P were chronically elevated in disease because of the continuing accumulation of misfolded PrP. This resulted in the sustained reduction of global protein synthesis rates in the brain. This chronic translational repression occurs in parallel to any possible neurotoxicity induced by PrP^Sc^ or soluble toxic species. The resulting decline in levels of key synaptic proteins exacerbates early synaptic failure and ultimately leads to terminal neuronal loss. Crucially, restoring protein synthesis rates by lowering levels of eIF2α‐P by either removing the source of the unfolded proteins (via RNAi‐mediated knockdown of the PrP) or reducing chronic UPR activation (by over‐expressing the phosphatase of eIF2α‐P, GADD34) prevented neurodegeneration and clinical disease (Moreno *et al*. 2012). This resulted in a recovery of synaptic protein levels, synaptic transmission and synapse number, and stimulated neuroprotection and markedly reduced spongiform degeneration in prion‐infected animals. The protection was seen despite ongoing prion replication and accumulation in mice with GADD34 over‐expression. Conversely, treating prion‐diseased mice with the small molecule salubrinal, which inhibits GADD34 induced dephosphorylation of eIF2α‐P, accelerated clinical disease and neuronal loss and shortened survival. Although mice over‐expressing the prion protein were used for the majority of these experiments, parallel experiments using mice with wild‐type levels of PrP also showed the same pattern of UPR activation, demonstrating that this effect is not an artifact of protein over‐expression in transgenic mice (Moreno *et al*. 2012). Thus, sustained eIF2α‐P‐mediated translational repression is a critical mediator of neurodegeneration in prion disease, and prevention of phosphorylation of eIF2α represents a valid therapeutic approach in neurodegenerative disease. This approach is likely to have the additional effect of improving learning and memory, which is of obvious benefit when treating dementia. Long‐term synaptic plasticity and memory formation relies on *de novo* protein synthesis, which is inhibited by chronic eIF2α phosphorylation (Costa‐Mattioli *et al*. 2009). ATF4 is also a repressor of cAMP response‐element binding protein‐mediated gene expression, which is essential for synaptic plasticity and learning (Chen *et al*. 2003). Unexpectedly, XBP1 has also recently been shown to regulate learning and memory independently of ER stress, via the activation of brain derived neurotrophic factor (Martinez *et al*. 2016).

**Table 1:** Current modulations and treatment strategies targeting the unfolded protein response

Therapy type	Examples	Mode of action	Disease model	References
Pharmacological	Trazodone	Increased ternary complex formation	Prion, FTD	Halliday *et al*., (2017)
	DBM	Increased ternary complex formation	Prion, FTD	Halliday *et al*., (2017)
	ISRIB	Stabilizes elF2B heterodimer	Prion	(Halliday *et al*., 2015; Sidrauski *et al*., 2015; Sekine *et al*. 2015)
	GSK2606414	PERK inhibition	Prion, FTD	Moreno *et al*., (2013; Radford *et al*. 2015)
	Salubrinal	Preventing elF2α dephosphorylation	PD, ALS	(Saxena *et al*., 2009; Colla *et al*., 2012)
	Guanabenz	Modulation of proteostasis	Charcot–Mare–Tooth IB	(D'Antonio *et al*., 2013; Crespillo‐Casado *et al*., 2017)
	Sephinl	Modulation of proteostasis	Charcot–Mare–Tooth IB, ALS	(Das *et al*., 2015; Crespillo‐Casado *et al*., 2017)
	TUDCA	Chemical chaperone	X‐linked ALD	Launay *et al*., (2017)
Genetic	PrP knockdown	Brain specific knockdown of PrP by RNAi	Prion	Moreno *et al*., (2012)
	GADD34 over‐expression	Focal increase in GADD34 via lentiviral injection	Prion	Moreno *et al*., (2012)
	PKR deletion	Genetic ablation	AD	Lourenco *et al*., (2013)
	PERK deletion	Genetic ablation	AD	Devi and Ohno, (2014)
	GCN2 deletion	Genetic ablation	AD	Ma *et al*., (2013)
	CRISPR/Cas9	Gene editing	Potentially multiple	Reviewed by Heidenreich and Zhang, (2016)
	Antisense oligonucleotides	Targeted mRNA interference	Potentially multiple	Reviewed by Evers *et al*., (2015)

**Figure 3:**
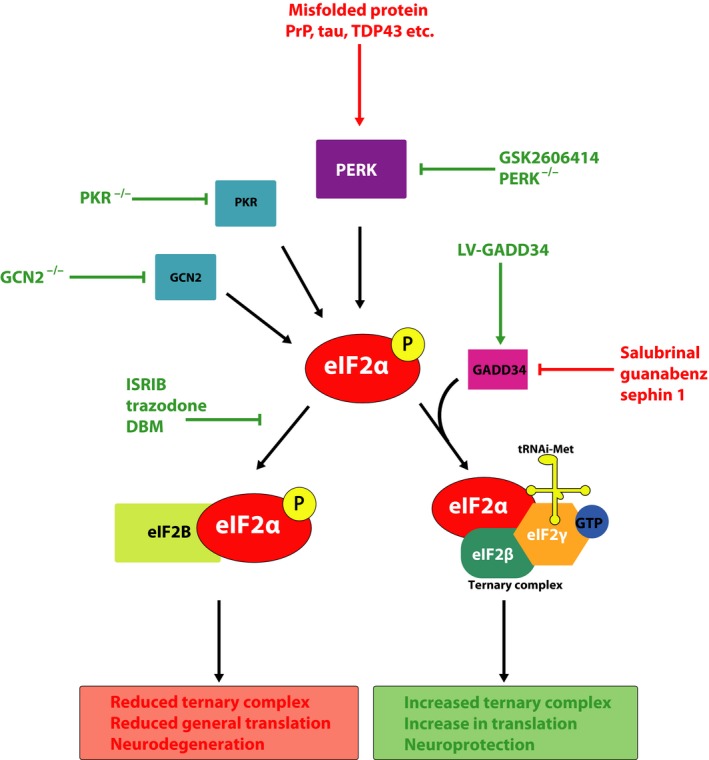
Modulating the unfolded protein response (UPR) to prevent neurodegeneration. Disease associated misfolded proteins activate protein kinase RNA (PKR) like ER kinase (PERK) signaling across the spectrum of neurodegenerative disease. Chronic translational repression is associated with neurodegeneration in several experimental model and human tissue samples. Genetic ablation of PERK, PKR and GCN2 improves learning and memory and neurodegenerative phenotypes, along with over‐expression of the eIF2α phosphatase GADD34. Small molecule inhibitors of PERK (GSK2606414), or compounds that promote ternary complex formation (ISRIB, trazodone and DBM) also restore translation and prevent neurodegeneration. Inhibitors of GADD34, such as salubrinal, guanabenz and sephin1 have been reported to enhance or protect against neurodegeneration depending on the experimental model used.

Other genetic approaches have also demonstrated a link between UPR activation and synaptic deficits seen in neurodegenerative disease models (Fig. 3). Genetic reduction of PERK or GCN2 lowers eIF2α‐P, and protects against impaired LTP and spatial memory in a model of AD (Ma *et al*. 2013; Trinh *et al*. 2014) and PERK repression in APP‐PS1 mice alleviates LTD impairment (Yang *et al*. 2016). Genetic deletion of PERK in 5xFAD mice lowers ATF4 expression and rescues memory deficits (Devi and Ohno 2014). Application of exogenous Aβ to wild‐type brain slices impairs induction of LTP but this effect is not seen in slices from PERK/GCN2 knockout mice (Ma *et al*. 2013). An interesting report has linked Aβ to brain inflammation, which converge onto PKR to cause synapse loss and memory impairment. This was ameliorated by PKR knockout, presumably through effects on eIF2α‐P levels (Lourenco *et al*. 2013). XBP1 signaling is also neuroprotective in models of disease; over‐expression of XBP1 driven by adeno‐associated viruses confers neuroprotection in models of PD, HD, glaucoma and models of sciatic nerve crush (Hu *et al*. 2012; Zuleta *et al*. 2012; Valdes *et al*. 2014; Onate *et al*. 2016). Studies in fly models also demonstrate that XBP1 can protect against Aβ and tau induced neurodegeneration (Loewen and Feany 2010; Casas‐Tinto *et al*. 2011). However, a recent study demonstrated that the genetic deletion of the RNase domain of IRE1 significantly reduced amyloid deposition and astrocyte activation in an AD model. IRE1 deficiency fully restored the learning and memory capacity of AD mice, associated with improved synaptic function and improved memory (Duran‐Aniotz *et al*. 2017). This suggests IRE1 and XBP1 may have divergent roles in AD.

## Targeting the PERK pathway for therapy pharmacologically

Genetic studies targeting PERK/eIF2α‐P signaling for neuroprotection have led to pharmacological proof of principle studies. The first of these used a small molecule inhibitor of the pathway (Fig. 3). Prion‐diseased mice were treated with the highly selective inhibitor of PERK, GSK2606414 (Axten *et al*. 2012). Treatment effectively reduced levels of PERK‐P and eIF2α‐P, despite ongoing prion replication, restoring vital protein synthesis rates in the brain (Moreno *et al*. 2013). As with genetic manipulation of the UPR, there was an absence of clinical signs of disease and marked neuroprotection throughout the brain. The beneficial effects were maintained in animals treated after behavioral signs had emerged. Unfortunately, effects on survival could not be measured as a result of pancreatic toxicity associated with GSK2606414, which resulted in weight loss and mild hyperglycemia, requiring termination of the experiment. Indeed, *Perk*‐/‐ mice display diabetes and greatly reduced lifespan, although *Perk+/‐* mice are indistinguishable from wild‐type mice except for a mild defect in glycemic control (Harding *et al*. 2001). However, the marked neuroprotective effect was extremely promising as proof of principle, particularly as it targeted a generic downstream process of protein misfolding and not PrP^Sc^, the specific pathogenic protein.

PERK‐mediated translational repression has also been identified as a key cause of neurodegeneration in a mouse model of FTD, which over‐expresses the human tau mutation P301L (Abisambra *et al*. 2013; Radford *et al*. 2015). Protein synthesis rates are again impaired in this model, alongside elevated levels of eIF2α‐P and ATF4 (Radford *et al*. 2015). The onset of eIF2α‐P‐mediated translational repression is closely associated with the onset of neurodegeneration, with tauP301L+ mice exhibiting hippocampal neuronal loss and memory impairment concurrently, mirroring what happens in prion‐diseased mice. Treatment with GSK2606414 again significantly reduced the levels of PERK‐P, eIF2α‐P and ATF4, restoring protein synthesis rates and protecting against further neuronal loss (Radford *et al*. 2015). Brain atrophy and clinical symptoms were also reduced after treatment. Interestingly, PERK inhibition also reduced the levels of phosphorylated tau. There appears to be a significant link between activation of both PERK and PKR, and the activation of glycogen synthase kinase‐3β (GSK3β) (Baltzis *et al*. 2007). Activation of PERK or PKR results in GSK3β activation, which leads to the phosphorylation of tau at disease relevant epitopes. PERK inhibition therefore acts via two mechanisms to protect against tau‐mediated neurodegeneration, by restoring vital protein synthesis rates and by decreasing the pathological phosphorylation of tau.

PERK inhibition has also been tested in fly models of sporadic ALS and PD. In the ALS model of disease, a progressive increase in the levels of eIF2α‐P was observed in *Drosophila* expressing TDP‐43 (Kim *et al*. 2014). After treatment with GSK2606414, TDP‐43‐expressing flies showed markedly lower levels of eIF2α‐P, with a dramatic reduction of TDP‐43‐induced climbing dysfunction and motor weakness. Similar results were observed in flies expressing PINK1 and parkin mutants; GSK2606414 treatment attenuated ER stress and conferred neuroprotection (Celardo *et al*. 2016).

These promising *in vivo* results from multiple laboratories in multiple models support the approach of reducing PERK signaling for treatment/prevention of these disorders in principle. However, the pancreatic side effects of direct – and highly effective – PERK inhibition must be overcome before translation into a clinical setting is possible. Yu *et al*. (2015), showed that GSK2606414 toxicity was mediated by an interferon response that is normally kept in check by PERK activation. Anti‐interferon therapy prevented the toxicity induced by GSK2606414, and such antibody‐based therapies are already in clinical trials for the treatment of inflammatory and autoimmune diseases (McBride *et al*. 2012; Higgs *et al*. 2014). Another approach is to restore translation rates by inhibiting signaling downstream of PERK‐P and eIF2α‐P. This was first made possible with the discovery of the interesting compound ISRIB (Sidrauski *et al*. 2013), which binds directly to eIF2B and allows ternary complex to be formed even in the presence of eIF2α‐P (Sekine *et al*. 2015; Sidrauski *et al*. 2015). It elicits these effects without affecting the levels of eIF2α phosphorylation. ISRIB improves memory in wild‐type mice (Sidrauski *et al*. 2013) and prevents neurodegeneration in prion‐diseased mice (Halliday *et al*. 2015). Crucially, ISRIB treatment is neuroprotective without pancreatic toxicity, because of ISRIB restoring protein synthesis partially (Halliday *et al*. 2015), rather than the complete restoration seen with GSK2606414 (Moreno *et al*. 2013). The partial restoration of protein synthesis rates is sufficient to protect neurons and increase the life span of ISRIB‐treated prion‐diseased mice, while allowing the pancreas to correctly handle physiological protein folding and ER stress. Fine‐tuning the extent of UPR inhibition and subsequent translational de‐repression is therefore able to uncouple neuroprotective effects from pancreatic toxicity. Unfortunately, ISRIB is too insoluble to become a human treatment in its own right, but demonstrates the efficacy of modulating downstream PERK signaling, but excitingly, recent work has uncovered two compounds that inhibit eIF2α‐P signaling in a similar manner to ISRIB, which, critically, are much better placed to be translated into human treatments for neurodegeneration (Halliday *et al*. 2017).

**Figure 4:**
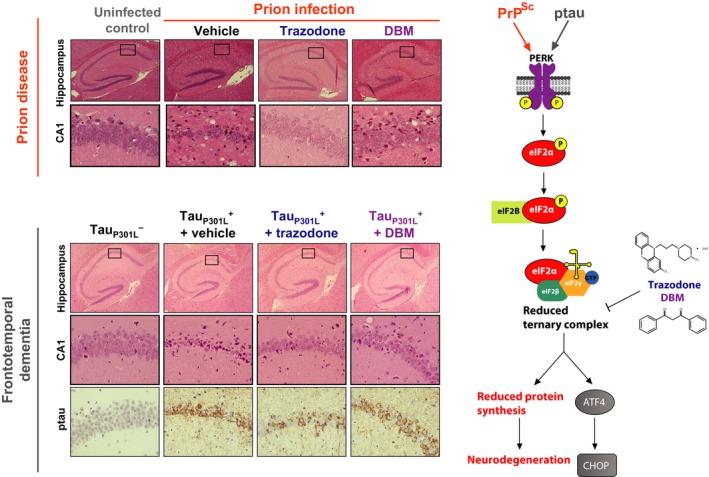
Repurposed drugs prevent neurodegeneration in models of prion and frontotemporal dementia (FTD). Two compounds, trazodone and DBM, recently uncovered in a screen for unfolded protein response (UPR) inhibitors prevent neurodegeneration in the prion and FTD mouse models of disease. Both compounds are effective when administered after synaptic dysfunction has begun. Trazodone also reduced phosphorylated tau aggregation in the FTD model. The compounds act downstream of eIF2α‐P to increase ternary complex levels. Adapted from (Halliday *et al*. 2017).

In this recent study, the authors performed a drug screen on a library of compounds comprised of approximately 75% already approved drugs in a *Caenorhabditis elegans* and cell‐based models of UPR overactivation. Two hits uncovered in this screen, the antidepressant trazodone and the naturally occurring compound dibenzoylmethane (DBM), were then tested in both the prion and FTD models used in previous experiments (Moreno *et al*. 2013; Halliday *et al*. 2015; Radford *et al*. 2015). Both compounds partially restored protein translation rates, extended lifespan, conferred neuroprotection and improved behavioral symptoms associated with these models, without any pancreatic toxicity (Halliday *et al*. 2017) (Fig. 4). In a similar manner to ISRIB, both compounds acted downstream of eIF2α‐P. They were found to act by restoring levels of ternary complex, although their exact binding sites were not determined (the protein translation restoring effects are believed to be divergent from their primary mechanisms of action). Interestingly, trazodone (but not DBM) was observed to lower phosphorylated tau levels in the FTD model. UPR activation induces tau phosphorylation via activation of GSK3β (Nijholt *et al*., 2013) and in agreement with this, inhibiting PERK using GSK2606414 also lowers tau phosphorylation (van der Harg *et al*. 2014; Radford *et al*. 2015). The reduction in phosphorylated tau after trazodone treatment is therefore likely because of its UPR inhibitory effects. However, as trazodone and DBM induced a similar degree of neuroprotection, it is likely the partial restoration of protein synthesis and reduction of the stress response downstream of eIF2α‐P is the primary driver of neuroprotection in both tauopathy and prion‐diseased mice. This further implicates the UPR as a central process in neurodegeneration. Trazodone, a licensed antidepressant is safely used in AD for the management of agitation and insomnia, albeit usually in advanced disease (McCleery *et al*. 2014), where benefit would be less likely as a result of the momentum of disease progression. DBM is a naturally occurring structural analog of curcumin, with widely reported anticancer properties (Khor *et al*. 2009), which has no known toxicity. Both compounds have the potential to be repurposed for neurodegenerative treatments.

## Other approaches to fine‐tuning PERK signaling

As the previously discussed, this work demonstrates the level of PERK signaling needs to be finely controlled to elicit neuroprotective effects without toxicity. The IRE1 and ATF6 arms of the UPR also up‐regulate chaperone proteins and induce protein degradation pathways that help with the disrupted proteostasis associated with neurodegeneration, so maintaining signaling through these branches while modulating PERK activity is desirable (Hetz and Mollereau 2014). Although PERK signaling is cytoprotective in response to mild ER stress, constitutive PERK activation can act as a pro‐death signal (Lin *et al*. 2007) and constitutive PERK activity has negative effects on both cell proliferation and viability (Lin *et al*. 2009). The switch from beneficial ATF4 signaling to induction of the pro‐apoptotic CHOP is also a point at which PERK signaling could be driven towards neuroprotective processes. The modulating effects of ISRIB on eIF2B and on trazodone, and DBM on ternary complex formation provide one such therapeutic target. As PERK activation is not affected by these compounds, the neuroprotective Nrf2 pathway will still be engaged (Joshi and Johnson 2012). Other possible points of modulation include p58IPK, calcineurin and Protein kinase B (PKB/AKT).

The Hsp40 family member p58IPK, which is up‐regulated in response to ER stress (Gupta *et al*. 2010), has been proposed to inhibit PERK through direct binding of its cytosolic kinase domain. This helps to ensure that eIF2α phosphorylation (and associated translational repression) as well as the increase in pro‐apoptotic CHOP, is transient following ER stress (van Huizen *et al*. 2003). However, it has been shown that p58IPK is localized to the ER lumen and is found in complex with BiP where it plays a role in enhancing ER protein folding capacity (Rutkowski *et al*. 2007). It is proposed to inhibit PERK by reaching sufficient quantities in the cytosol during ER stress as a result of its inefficient ER targeting sequence to interfere with its kinase domain (Rutkowski *et al*. 2007).

The calcium and calmodulin‐dependent phosphatase calcineurin was identified as another direct modulator of PERK signaling (Bollo *et al*. 2010). Calcineurin binds to PERK's cytosolic kinase domain and promotes autophosphorylation, which in turn enhances PERK signaling and the translational attenuation resulting from it. How calcineurin, a phosphatase, could promote the autophosphorylation and activation of PERK, is paradoxical, but it is speculated that calcineurin may play a role in stabilizing the PERK protein (Donnelly *et al*. 2013). Calcineurin inhibitors are already used in the clinic as immunosuppressors, and interestingly there are several reports of neuroprotective effects after calcineurin inhibitor administration (reviewed in (Kipanyula *et al*. 2016).

PERK is also a direct target of AKT, which regulates PERK signaling through inhibitory phosphorylation (Mounir *et al*. 2011). AKT inhibits PERK signaling, and promotes survival under conditions of ER stress by lowering eIF2α phosphorylation and ATF4 and CHOP expression. Inhibition of AKT sensitizes cells to ER stress‐induced apoptosis (Hu *et al*. 2004). This demonstrates that although PERK signaling is essentially pro‐survival in response to ER stress, its activation must be tightly regulated; there appears to be an optimal level of PERK signaling beyond which PERK signaling becomes toxic because of CHOP induction and sustained translational repression. A small molecule activator of AKT, SC79, has been shown to protect against ischemia induced cell death (Jo *et al*. 2012).

## Conclusions

As discussed, constitutive PERK signaling has been shown to promote cell death by apoptosis (Lin *et al*. 2009), and PERK signaling in the absence of the other UPR components has been suggested to represent the terminal, pro‐apoptotic phase of the UPR, that responds to prolonged ER stress (Lin *et al*. 2007). Directly inhibiting PERK is markedly neuroprotective (Moreno *et al*. 2013; van der Harg *et al*. 2014; Kim *et al*. 2014; Radford *et al*. 2015; Celardo *et al*. 2016), but causes pancreatic toxicity if this inhibition is too strong (Moreno *et al*. 2013; Halliday *et al*. 2015). However, recent reports have demonstrated that it is possible to fine‐tune the level of inhibition to confer neuroprotection without pancreatic toxicity (Halliday *et al*. 2015, 2017), as discussed above. These reports validate the UPR as an important target in neurodegeneration and demonstrate that generic cellular processes can be leveraged to provide benefits without inhibiting upstream protein misfolding events. Excitingly, this approach would provide treatment across the spectrum of the protein misfolding neurodegenerative diseases, as it does not focus on each individual disease‐specific protein. There are examples of neurological disorders, including the rare SOD1 G85R ALS variant, and the rare peripheral neuropathy Charcot–Marie–Tooth disease type 1b, however, where signaling via the PERK branch of the UPR is not deleterious (Saxena *et al*. 2009; Wang *et al*. 2011; Colla *et al*. 2012; D'Antonio *et al*. 2013; Das *et al*. 2015). Indeed, in these models, prolonging eIF2α phosphorylation is protective, although the mechanism of action of the compounds used in these experiments has recently been questioned (Crespillo‐Casado *et al*. 2017). These results are not necessarily contradictory, however, because of the different models used and the intracellular locations of the misfolded protein build‐up, as well as the timings of treatments, which may all determine outcome (discussed in more detail in (Hetz and Mollereau 2014; Scheper and Hoozemans 2015; Smith and Mallucci 2016). Another unresolved issue is how the misfolded proteins are activating the UPR in several of these diseases, as canonical UPR activation is dependent on PERK, IRE1 and ATF6 detecting misfolded proteins in the ER lumen, while misfolded protein aggregates are generally cytoplasmic or extracellular. PERK can be activated via ischemia (Sanderson *et al*. 2010), which hints there are other mechanisms of UPR activation. Despite these caveats, partial inhibition of the PERK branch signaling UPR represents an increasingly exciting therapeutic target for the treatment of neurodegenerative diseases. The challenge now is to translate these targets into effective human therapeutics, possibly by using the pharmacological agents discussed above, or by exploiting the promise of CRISPR‐Cas9 gene editing or antisense oligonucleotides (Evers *et al*. 2015; Heidenreich and Zhang 2016).
